# Knowledge about neonatal danger signs and associated factors among mothers of children aged 0–12 months in a rural county, Southwest of China: a cross-sectional study

**DOI:** 10.1186/s12884-022-04592-4

**Published:** 2022-04-21

**Authors:** Jingxin Zhou, Wenzhe Hua, Qiaomu Zheng, Qin Cai, Xi Zhang, Liping Jiang

**Affiliations:** 1 Department of Neonatal Intensive Care Unit, Xinhua Hospital Affiliated To Shanghai Jiao Tong University School of Medicine, Shanghai, China; 2 Shanghai Jiao Tong University School of Nursing, Shanghai, China; 3 Children’s Hospital of Shanghai Jiao Tong University, Shanghai, China; 4 Clinical Research Unit, Xin Hua Hospital Affiliated To Shanghai Jiao Tong University School of Medicine, Shanghai, China; 5 Department of Nursing, Xinhua Hospital Affiliated To Shanghai Jiao Tong University School of Medicine, Shanghai, China

**Keywords:** Knowledge, Neonatal danger signs, Minority ethnic, Low-income, Rural China

## Abstract

**Background:**

Delay in care seeking is one of the causes for neonatal death. Mothers’ knowledge of neonatal danger signs is imperative to promote early recognition of neonatal illness and reduce the delay in care seeking. Currently, no study has been conducted on the knowledge about neonatal danger signs in China, especially in economically less developed areas. This study aimed to examine the knowledge of neonatal danger signs and risk factors of poor knowledge among mothers in a rural county of southwest of China.

**Methods:**

A cross-sectional study was conducted in Wenshan, a rural county of southwest of China. A total of 112 respondents were included from November 2020 to February 2021 among women who had babies aged 0–12 months and brought their babies to health care centers for immunization within the study period. A questionnaire with 18-item key neonatal danger signs was used to measure their knowledge about these signs. Mothers who scored above average were considered to have relatively good knowledge whereas those who scored below average were considered to have relatively poor knowledge. Independent predictors of mothers’ knowledge were identified by multivariable logistic regression analysis.

**Results:**

The mean knowledge score of neonatal danger signs of mothers was 18.1 (SD = 8.6). Fifty-eight percentage of mothers (65/112) had poor knowledge of neonatal danger signs. Danger signs of “bluish or pale skin”, “chest indrawing”, and “convulsion” were mostly recognized, whereas danger signs of “not able to feed since birth, or stopped feeding well”, “excessive crying” and “eyes draining pus” were recognized poorly. Less than four antenatal visits [AOR = 4.348], younger than 25 years old [AOR = 3.839], ethnic minority [AOR = 3.956] and family financial difficulty [AOR = 4.944] were significant indicators of relatively poor knowledge.

**Conclusions:**

Mothers’ knowledge about neonatal danger signs in rural China is poor even though the coverage of maternal and child health care services are expanded. Existing efforts should be enhanced for antenatal care visits, avoiding early marriage as well as early childbearing. More attention should be paid to low-income ethnic minority mothers. Educating and training should be strengthened for danger signs, especially those who are predicted to have insufficient knowledge.

**Supplementary Information:**

The online version contains supplementary material available at 10.1186/s12884-022-04592-4.

## Background

Neonatal danger signs were proposed by World Health Organization (WHO) and United Nations International Children’s Emergency Fund(UNICEF) [1], which indicates newborns being at high risk of illness and death. Any of these signs’ existence needs early detection. Timely and adequate care-seeking is crucial to improve neonatal health and survival. Failure to seek medical care might be related to caregivers’ inadequate knowledge of neonatal danger signs [2]. Good knowledge of these signs plays a vital role in reducing mortality [3]. The levels of knowledge of danger signs vary between different countries and regions. In Uganda, India, Ethiopia and Nigeria, the percentage of caregivers with poor knowledge of neonatal danger signs range from 38 to 85% [4–7].

The neonatal period is one of the critical periods for a child’s survival. Every year over 2.4 million babies die during the neonatal period globally [8]. Although the average global rate of neonatal mortality was 17 deaths per 1000 live births in 2019, down by 54% from 37 deaths per 1000 in 1990, the neonatal mortality declined slower than mortality of children aged between 1–11 months and 1–4 years [9, 10]. The vast majority of newborn deaths occur in low and middle-income developing countries [10].

China, with 64 thousand neonatal deaths occurred by the 2019 report, is still one of the top 10 countries with the highest number of newborn deaths in 2019 and ranks 15 in Asia in terms of neonatal mortality [8, 11]. The neonatal mortality rate declined from 24.9 deaths per 1000 live births in 1990 to 4.5 deaths per 1000 live births in 2017 [12]. China has made a significant progress in reducing the under-five mortality rates (Millennium Development Goal 4) by 2/3 from 1990 levels [13]. Yet, in economically less developed regions, the risk of neonatal death is higher compared with developed ones. In rural areas, newborns have the least chance of receiving medical care before death among the population [14, 15]. Among deaths of children under 5 years of age, two-thirds of them are newborns, and there is still a significant gap between rural and urban areas [16, 17]. In southwest of China, the neonatal mortality rate, infant mortality rate and under-5 mortality rate achieved a sustainable and substantial decrease from 2000 to 2014 after years of efforts [17]. However, we can still find that 47.53% of children died at home and 34.53% of children didn’t even receive any medical care before death in southwest of China from a research analyzing the deaths of children under the age of five by verbal and social autopsy [15]. Relevant reports are lacking in recent years on the improvement and current situation in southwest of China.

Research on danger signs in newborns has received less attention in China, especially in poor regions. To the best of our knowledge, there were no studies to assess the knowledge about neonatal danger signs in rual China. In this study, we chose mothers as the respondents because they were considered primary caregivers of newborns in local. The present study aimed to assess mothers’ overall level of knowledge of neonatal danger signs in Wenshan, a rural county of southwest of China, and to identify what danger signs were poorly recognized, and to explore associating social-economic factors with knowledge of neonatal danger signs.

## Methods

### Study setting

This study was carried out in Wenshan, a rural county of southwest of China. The total registered population is 513,000. More than 10 ethnic groups live there, many of whom are ethnic minorities such as Zhuang, Hmong, Yi, Hui, et al., while the majority is Han ethnicity in China. More than 97% of the region is mountainous. White Paper on Human Poverty Reduction in China shows that the per capita disposable income of China of rural residents in poor areas increased from 6,079 yuan in 2013 to 12,588 yuan in 2020 [18]. The 2020 data showed that the average disposable income of Wenshan of permanent rural residents was ¥13,321 [19]. With the help of China’s poverty alleviation program, the county was officially removed from the list of Chinese poverty counties on May 17, 2020. The county was nicknamed the hometown of a plant of medical value because the trade of the plant was the driving force of the city’s economic development. Despite of the rich natural resources, an unignorable proportion of residents still lives in rural poverty.

### Study design and participant characteristics

This was a cross-sectional study using multistage sampling. The study was conducted in four health care centers in Wenshan, a rural county of southwest of China from November 2020 to February 2021. Wenshan has a total of 7 towns. According to the economic level on the budget of the financial revenue of each town, we chose the town with the highest and lowest budget and 2 towns of the remained five. In the second stage, one health care center was selected from each town using convenience sampling. We used simple random sampling to recruit our respondents. Only women who had babies aged 0–12 months and brought their babies to health care centers for immunization within the study period were recruited. In China, a newborn baby needs to be vaccinated with the free vaccines designated by the state every month within the first six months, as well as at 8 and 9 months after birth. Mothers should be permanent residents of the county and provided written informed consent to participate in this study.

### Sample size determination and sampling procedure

The sample size was calculated adopting the same method from another study [7]. Based on the birth rate of 1.34% and a projected population in the county [20] with an anticipated non-response rate of 10% and a 5% margin of error, a minimum of 23 mothers were expected to be enrolled in this study. For each of the four communities visited, our sample size requires a minimum of 92 mothers. Mothers who met the inclusion criteria and informed consent to participate were recruited. Finally, a total of 112 mothers were included from all four health centers. Mothers with any of mentally and physically problems were excluded.

### Measurement and data collection procedure

Data were collected using a structured questionnaire by four trained data collectors who are expertise in pediatrics and fluent in the local dialects. The data collectors are responsible for the completeness and consistency of data at the site. The questionnaire includes variables related to socio-demographic characteristics such as age, educational status, occupation status and healthcare service utilization associated factors of the mother. Moreover, 18-item key neonatal danger signs were considered to measure the knowledge of mothers on these signs. Eleven of the items listed in the questionnaire were outlined by WHO/UNICEF [1] (items 9,10,11 were derived from items 9 of WHO/UNICEF, which refers to three different symptoms)while another 7 items were also regarded as the potentially severe conditions in local hospitals. The signs were: 1) Not able to feed since birth, or stopped feeding well; 2) Convulsion; 3) Fast breathing: 60 breaths or more in one minute; 4) Chest indrawing; 5) High temperature: 37.5℃ or more; 6)Very low temperature: 35.4℃ or less; 7)Movement only when stimulated, or no movement even on stimulation; 8)Yellow soles; 9)Umbilicus red or draining pus; 10)Skin boils; 11)Eyes draining pus; 12)Birth within 37 weeks or birth weight < 2500 g; 13)Bluish or pale skin; 14)Diarrhea; 15)Excessive crying; 16)Blood in stool; 17)Abdominal distension; 18)Frequent vomiting. The internal reliability of the questionnaire was checked by computing Cronbach’s alpha (0.928).

### Operational definition

Likert 5 grading method was adopted in the knowledge score for neonatal danger signs. We can find the same method from another study [21]. Each sign was regarded as dangerous and the mothers were asked to choose the degree to how much they agreed. Five options were “strongly agree”,” agree”,” not necessarily”, “disagree” and “strongly disagree” respectively. Two points were assigned to “strongly agree”, one point was assigned to “agree”, and the rest were not scored. Each item was scored between 0 to 2 points. Knowledge score was computed by adding all the values of each item with a minimum score of 0 and a maximum of 36 (0 when a mother chose “not necessarily”, “disagree” or “strongly disagree” of all the neonatal danger signs). Two categories were defined for knowledge of neonatal danger signs. All women who scored greater than or equal to the average were considered knowledgeable while the rest were considered poor, defined similarly as in a previous study [22].

### Data quality control methods

A pretested, structured, and interviewer-administered Chinese questionnaire was used in this study. The 18 items of danger signs were translated to Mandarin and then back-translated by language expertise. Questionnaires were asked in Mandarin but always interpreted using local languages for mothers to understand better as conducted in other studies [13]. Data collection was conducted by data collectors who had received an intensive 2-day training course (one day of theoretical training and the other day of practical training) from the supervisor. The training focused on the purposes of this study and the meaning of every single question. Before the actual data collection, the questionnaire was pilot on 25 women in the county to evaluate the validity of the measurement and reaction of the participants to the questions. Subtle corrections on the measurement were made accordingly. During data collection, the data collectors were closely monitored and guided by the supervisor for the complete and appropriate collection of the data. The data collectors offered feedback to the study researcher if necessary. The data was entered using SPSS by double-checking for validation purpose.

### Data processing and analysis

The data collected was coded, entered and analyzed in SPSS 25.0. Frequencies, proportion and summary statistics were used to describe the respondents concerning relevant variables. The score of the neonatal danger signs was represented as means and standard deviation (SD). Binary logistic regression was used to assess the association between the knowledge of mothers about neonatal danger signs and individual variables. Variables with a *p*-value < 0.25 in the bivariable analyses were included in the multivariable logistic regression model to investigate statistically significant predictors of mothers’ knowledge of danger signs. Variables with a *p*-value < 0.05 from multivariable logistic regression were regarded as statistically significant estimated by adjusted odds ratio (AOR) along with 95% confidence interval (CI). Hoshmer-Lemeshow statistic and Omnibus tests were then used to check the model fitness.

### Ethical consideration

The Ethics Committee of local People’s Hospital rendered ethical approval to this study. The respondents were required to sign an informed consent form before the questionnaire section. Due to the low level of education in the region, the data collectors explained this study before verbal voluntary consent was obtained from mothers. If the mothers were unable to read and write, the study was conducted by face-to-face interview and the questionnaires were then filled by data collectors according to the mothers’ verbal answers. Parental consent was required if the participants were under 18 years old. The participants were told that the information they provide would be kept confidential. There was no physical harm or intervention throughout the entire study and they could withdraw from the study at any time. After the survey, mothers could ask any questions about the study and were provided with the education of neonatal danger signs if they wanted.

## Results

### Socio-demographic factors

A total of 116 mothers were selected to participate, 112 of them completed the questionnaire (response rate 96.5%). The mean age of the respondents was 27.2 (SD ± 5.84) years. The minimum age of study participants in our study was 15 years old while the maximum age was 41 years old. Among them, 42 (37.5%) mothers were between the age of 15–25 years and more than two-thirds of mothers (68.8%) were unemployed. Only 85.7% of the respondents were married. More than half of the participants (54.5%) had at least two children. The majority of them had middle (29.5%) and high school (25.9%) as their highest educational attainment while 27 (24.1%) had college or above education, and a minority (5.4%) were illiterate or close to illiterate. Most women (67.0%) were from ethnic minorities. Nearly half of mothers were in family financial difficulty (Table 1).

**Table 1:** Social-demographic of mothers

Items		Frequecy	Percentage
Ages(years)	< 25	42	37.5
	≥ 25	70	62.5
Educational level	Illiteracy or little literacy	6	5.4
	Primary school (1–5 grades)	17	15.2
	Middle school (6–9 grades)	33	29.5
	High school (10–12 grades)	29	25.9
	College or above (12–14 grades)	27	24.1
Occupation	employed	35	31.3
	unemployed	77	68.7
Ethnicity	Han	37	33
	Ethnic minority	75	67.0
Marital status	Married	96	85.7
	Unmarried or others	16	14.3
Number of children	1	51	45.5
	2	44	39.3
	> 2	17	15.2
Gender of neonate	Male	62	55.4
	Female	50	44.6
Family financial difficulty	Yes	53	47.3
	No	59	52.7

### Mothers’ healthcare service utilization related factors

Three fourths (75%) of the mothers had more than four antenatal care (ANC) visits. Most of the mothers (97.3%) delivered in a health institution, 6.3% of the mothers experienced premature birth, and 34.8% experienced hospitalization of children in the neonatal period, and 93 (83%) had at least one postnatal care (PNC) follow-up visit. Three-fourths of mothers (75.9%) gave birth vaginally. When the mothers were asked whether they had been educated about the neonatal danger signs, the majority (69.6%) of them answered never (Table 2).

**Table 2:** Healthcare service utilization related factors of mothers in Wenshan, a rural county, Southwest of China, *n* = 112

Variables		Frequecy (n)	Percentage (%)
Number of ANC visit	≥ 4	84	75
	< 4	28	25
Delivery place	Health institution	109	97.3
	Home	3	2.7
Delivery method	Vaginal delivery	85	75.9
	Cesarean delivery	27	24.1
Number of education about neonatal danger signs	0	78	69.6
	1	22	19.6
	2	7	6.3
	≥ 2	5	4.5
Experience of premature birth	Yes	7	6.3
	No	105	93.8
Experience of hospitalization of children in neonatal period	Yes	39	34.8
	No	73	65.2
PNC visit	Yes	93	83
	No	19	17

*ANC* antenatal care, *PNC* postnatal care

### Score of knowledge of mothers about neonatal danger signs

**Fig. 1:**
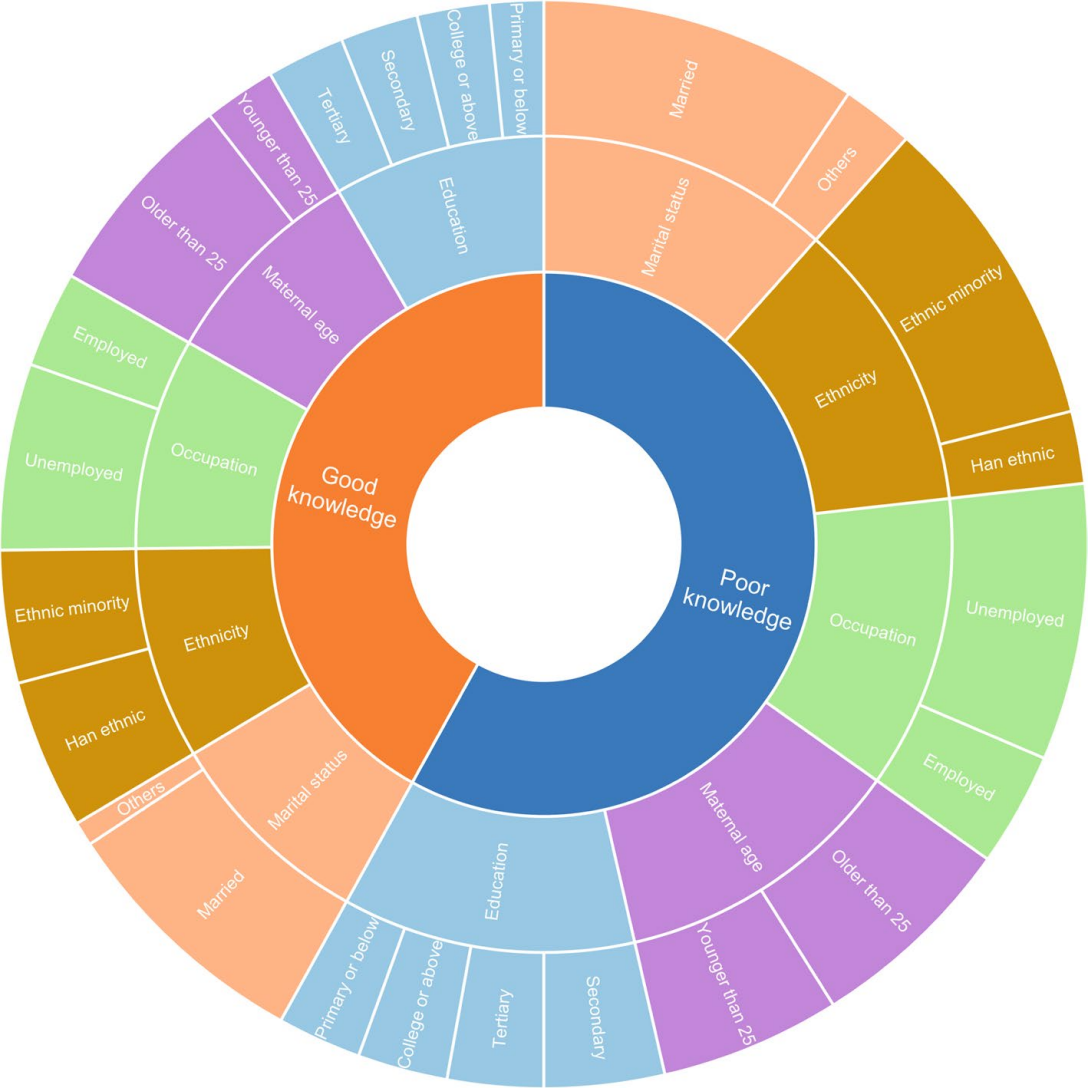
The level of knowledge about neonatal danger signs among mothers and the composition of maternal characteristic

Table 3 shows the knowledge of mothers about danger signs. The most recognized danger signs by the mothers were “bluish or pale skin” (1.41 ± 0.65), “chest indrawing” (1.41 ± 0.62) and “convulsion” (1.33 ± 0.69). The least recognized danger signs were “not able to feed since birth, or stopped feeding well” (0.47 ± 0.64), “excessive crying” (0.72 ± 0.74) and “eyes draining pus” (0.79 ± 0.71). The average knowledge score of neonatal danger signs of mothers was (18.07 ± 8.58). Knowledge score was categorized as good (above the average score) and poor (below the average score). Based on the score, 47 (42%) of the mothers had good knowledge, whereas 65 (58%) had poor knowledge. The knowledge level about neonatal danger signs among mothers and the composition of maternal characteristics are shown in the figure below (Fig. 1).

**Table 3:** The knowledge about neonatal danger signs

Items	Score	Ranks
Not able to feed since birth,or stopped feeding well	0.47 ± 0.64	18
Excessive crying	0.72 ± 0.74	17
Eyes draining pus	0.79 ± 0.71	16
Diarrhea	0.80 ± 0.75	15
Yellow soles	0.81 ± 0.66	14
Abdominal distension	0.86 ± 0.75	13
Birth within 37 weeks or birth weight < 2500 g	0.89 ± 0.80	12
Frequent vomiting	0.91 ± 0.73	11
Fast breathing: 60 breaths or more in one minute	1.01 ± 0.70	9
Skin boils	1.01 ± 0.72	9
Very low temperature:35.4℃ or less	1.03 ± 0.75	8
Umbilicus red or draining pus	1.05 ± 0.71	7
High temperature: 37.5℃ or more	1.11 ± 0.73	6
Movement only when stimulated, or no movement even on stimulation	1.17 ± 0.76	5
Blood in stool	1.27 ± 0.68	4
Convulsion	1.33 ± 0.69	3
Chest indrawing	1.41 ± 0.62	1
Bluish or pale skin	1.41 ± 0.65	1

### Factors associated with mothers’ knowledge about neonatal danger signs

Using binary logistic regression model, six variables were associated with mothers’ knowledge of neonatal danger signs from bivariable analysis (*p*-value < 0.25). These were marital status, the number of ANC visits, maternal age, maternal ethnicity, family financial difficulty and whether the baby had hospital experience during the neonatal period.

Based on multivariable analysis, four of these variables were significantly associated with mothers’ knowledge about neonatal danger signs (*p*-value < 0.05). There were the number of ANC visits, maternal age, maternal ethnicity and family financial difficulty.

In this study, mothers who attended less than 4 ANC visits during pregnancy were 4.3 times more likely to have low-level knowledge of neonatal danger signs as compared to those who had attended more than 4 ANC visits [AOR = 4.348, 95%CI (1.299–14.558)]. Mothers younger than 25 years old were 3.8 times more likely to have poor knowledge comparing with mothers older than 25 [AOR = 3.839, 95%CI (1.359–10.845)]. Maternal ethnic group also was a significant factor since those ethnic minority mothers were 3.9 times more likely to have poor knowledge of neonatal danger signs as compared to mothers with Han ethnicity [AOR = 3.956, 95%CI (1.512–10.354)]. Besides, those mothers with family financial difficulty were 4.9 times more likely to have poor knowledge about danger signs than the mothers with no [AOR = 4.944, 95%CI (1.888–12.944)] (Table 4).

**Table 4:** Determinants of mothers’ poor knowledge about neonatal danger signs in Wenshan, a rural county, Southwest of China, (*n* = 112)

Factors	Knowledge about neonatal danger signs			95%CI	
	Good	Poor	Mean score	Crude OR	Adjusted OR
	n (%)	n (%)	$\bar{\mathbf{x}}$± S		
Marital status					
Married	43(38.4%)	53(47.3%)	18.59 ± 8.93	1	1
Unmarried or others	4(3.6%)	12(10.7%)	14.94 ± 5.32	2.434(0.732–8.089)	0.628(0.148–2.658)
The Number of ANC visit					
< 4	6(5.3)	22(19.7)	13.68 ± 8.93	3.496(1.288–9.493)	4.348(1.299–14.558) *
≥ 4	41(36.6)	43(38.4)	19.54 ± 8.00	1	1
Maternal age (years)					
< 25	12(10.7)	30(26.8)	16.36 ± 7.13	2.500(1.104–5.660)	3.839(1.359–10.845)*
≥ 25	35(31.3)	35(31.3)	19.10 ± 9.25	1	1
Maternal ethnic group					
Han	25(22.3)	12(10.7)	22.76 ± 7.18	1	1
Ethnic minority	22(19.6)	53(47.3)	15.76 ± 8.31	5.019(2.147–11.731)	3.956(1.512–10.354) **
Family financial difficulty					
Yes	13(11.6)	40(35.7)	14.74 ± 8.37	4.185(1.859–9.419)	4.944(1.888–12.944) ***
No	34(30.4)	25(22.3)	21.07 ± 7.67	1	1
Experience of hospitalization of children in neonatal period					
Yes	13(11.6)	26(23.2)	17.15 ± 8.30	1	1
No	34(30.4)	39(34.8)	18.56 ± 8.75	0.574(0.255–1.288)	0.526(0.191–1.447)

^*^ *p* < 0.05, ** *p* ≤ 0.01,***p ≤ 0.001

## Discussion

Reducing neonatal morbidity and mortality requires immediate caregivers’ recognition of neonatal danger signs and visits to the nearby health institution for medical care. In this study, 47 (42%) of the mothers had good knowledge, whereas 65 (58%) had poor knowledge, which is consistent with the report of Alex-Hart in Nigeria [23], Degefa in southern Ethiopia [6] and Guta in Dire Dawa of Ethiopia [24]. Our results were also consistent with a systematic review and meta-analysis that reported that the overall pooled prevalence of mothers’ knowledge of neonatal danger signs was 40.7% [25]. In this study, the proportion of knowledgeable mothers about danger signs was higher than the studies conducted in North West of Ethiopia (18.2%) [26], Kenya (15.5%) [27], 4 regions of Ethiopia (29.3%) [28], Ambo town of central Ethiopia (20.3%) [29] and northeast Ethiopia (28.2%) [30]. But the proportion was lower than the findings of mothers attending public health institutions of Mekelle city [31]. From this study, cultural variations among participants and differences in health care services delivery might have contributed to the differences in mothers’ knowledge about neonatal danger signs. Moreover, methodological differences, classification criteria differences, variations in time and study setting were the important reasons that contributed to the different results.

The two most commonly known danger signs were “Bluish or pale skin” (1.41 ± 0.65) and “chest indrawing” (1.41 ± 0.62). These are visible and obvious signs difficult to be missed. In a study reported by Sandberg in Uganda, “difficulty breathing” was recognized by a proportion (30%) of mothers by spontaneous response [12]. “Bluish or pale skin” was the most common non-WHO-listed danger signs recognized by mothers in this study. An Indian research indicated that though not being recognized by WHO, some of the signs should be considered in danger signs list in developing countries as the signs are indicators of major causes of morbidity and mortality [1].

In the present study, other signs such as “excessive crying” and “eyes draining pus”, “diarrhea”, and” yellow soles” were all poorly known. Signs of “not able to feed since birth, or stopped feeding well” got the lowest score among the 18 signs. In a study conducted by Khadduri et al., “difficulty feeding” was also identified as the least recognizable danger signs [32]. The lack of awareness of “difficulty feeding” is the cause for concern in many situations because it is a condition life-threatening for newborns. In contrast to the findings of Ekwochi’s study, which mentioned “excessive crying” as a common non-WHO recognized neonatal danger signs [7], we found this sign was less recognized than other signs in this study. These two signs could be unspecific signals rendering the mother unable to judge correctly whether the newborn is really sick. This difference may be due to mothers’ different perceptions of the severity of the problems. It can be assumed that poor knowledge of these danger signs may lead to delay in seeking appropriate medical care. Knowledge of danger signs is crucial to appropriate and timely care seeking behavior in undeveloped countries [33, 34]. Educating should be strengthened on neonatal danger signs especially for items with poor scores.

Our study revealed that the number of ANC visits had a significant positive association with mothers’ knowledge about danger signs, which was consistent with the study conducted by Zaman in rural Bangladesh and Bayih in north-central Ethiopia [35, 36]. This could be reasonably explained by the fact that those who attended ANC visits frequently are more likely to acquire the knowledge about danger signs. Community-based interventions integrating strategies of counseling and home visiting have been proved to reduce fetal and neonatal mortality [37]. In this study, one-fourth of mothers attended the ANC visits less than 4, which shows they did not paying enough attention to prenatal examination. Efforts should be made to support pregnant mothers to attend ANC visits at the recommended frequencies.

Maternal age below 25 years old was found to be associated with increased odds of poor knowledge about neonatal danger signs. Similarly, the fact that younger mothers had lower knowledge of neonatal danger signs was also reported by another study conducted by Jemberia et al., which showed that caregivers older than 18 years old were 33% more aware of the neonatal danger signs than those younger than 18 [22]. Another study found that all women aged < 18 years had poor knowledge about neonatal danger signs [38]. In our study, 62% of mothers younger than 25 were primiparous, whom might be less experienced in caring for newborns. The youngest mother was only 15 years old while she was supposed to be getting married after reaching 20. The whole society should raise efforts to awake teenagers to avoid early marriage and early childbearing.

Ethnic minorities make up 59.6% of the population in the county. The ethnic minorities accumulated rich and valuable medical experience in the etiology, nomenclature, classification and treatment of diseases, building up strong cultural and religious beliefs [39]. However, in this study, the mean score among Han mothers about neonatal danger signs was 22.76 ± 7.18 while that of ethnic minorities was only 15.76 ± 8.31. Mothers of ethnic minorities were 3.9 times more likely to possess poor knowledge about neonatal danger signs. This may be due to the fact that the ethnic minorities have a unique medical theory system. Ethnicity’s ancient primitive medicine culture was passed down through primitive myth and legend [40]. The ethnic minorities live in a wide area with scattered villages and remote mountainous in southwest of China. Due to inconvenient transportation and poor information, ethnic minorities medicine has been passed down orally for a long time. In a qualitative study conducted by our team (yet to be published), we found ethnic minorities mothers self-medicated with herbs remedies for some diseases, such as neonatal jaundice, fever, diarrhea, etc. Herbal medicine is rich in the southwest of China, and the ethnic minorities people will take advantage of this unique natural resources by going up to the mountains to gather herbs. Cultural and religious factors have been reported as the main barriers influence caregivers’ perception of illness and care seeking behavior [41, 42]. In addition, the low level of knowledge may be due to the fact that mothers of ethnic minorities generally married and gave birth before the age of 20.

Mothers with family financial difficulty were 4.9 times more likely to be unaware of danger signs than those mothers with no. This finding was consistent with a study performed in rural Bangladesh [43]. Mothers in low-income settings may have limited recognition of danger signs. The consistency may be due to the fact that mothers who had financial difficulty were less likely to have higher education. On the contrary, mothers with financially secure are easier to get access to knowledge in different ways, such as mobile phones, the Internet and social network. A similar study reported by Anmut et al. [44] that women with higher family income were 56% more knowledgeable about neonatal danger signs than those with low family income.

Previous studies confirmed that knowledge of neonatal danger signs was associated with caregivers’ educational level [6, 30, 45]. Interestingly, educational background was not a significant factor in this study. An assumption might be the overall educational level of mothers was relatively low, which didn’t give educated mothers an advantage.

### Strengths and limitations of the study

In this study, we used Likert 5 grading method and gave the corresponding score value, which makes us clearer how mothers perceive the different danger signs while some studies used unprompted answers to assess mothers’ recognition of the danger signs [5, 29, 30, 44].

This study had several limitations. First, the respondents were mothers who had babies aged 0–12 months, thus the results may be affected by recall bias, and the mothers may fail to differentiate the period between the neonatal and postneonatal period. Second, though we had data quality control measures, the findings of our study might also be influenced by data collectors’ bias. Third, despite adjustments for socio-demographic factors, mothers’ healthcare service utilization related factors, due to the limitation of cross-sectional study design, there may be unidentified confounding variables. Fourth, there were three other towns that were not included in our study site, four health care centers may not be representative of all regions. The small sample size is also one of our limitations due to the difficulties in our process of data collecting in these areas. Besides, cluster effect may not be considered due to a limited sample size. In future studies, we plan to conduct more qualitative studies for further casual inference in more centers.

## Conclusions

The results of this study reveal an urgent need of educational efforts to improve the knowledge about neonatal danger signs. Particularly, maternal’s ANC visits, avoidance of early marriage and early childbearing. On the other hand, we should pay more attention to ethnic minority mothers and mothers who have family financial difficulty. Educating and training should be strengthened for danger signs with poor scores.

## Supplementary Information

**Additional file 1.**

## Data Availability

The data analyzed in this study will be available upon reasonable request from the corresponding author.

## Abbreviations

AOR: Adjusted odds ratio
CI: Confidence interval
SPSS: Statistical package for social science
ANC: Antenatal care
PNC: Postnatal care
