# Supplementary material for: Knowledge about neonatal danger signs and associated factors among mothers of children aged 0–12 months in a rural county, Southwest of China: a cross-sectional study
Source: BMC Pregnancy Childbirth. 2022 Apr 21;22:346. doi: 10.1186/s12884-022-04592-4 (PMC9026920; doi:10.1186/s12884-022-04592-4)
Supplement: Supplementary file 1 — Additional file 1. [file 12884_2022_4592_MOESM1_ESM.docx]

Questionnaire of knowledge about neonatal danger signs

Please select the best answer to the following items and tick the appropriate box

| The serial number | Items | Strongly agree | Agree | Not necessarily | disagree | Strongly disgree |
| --- | --- | --- | --- | --- | --- | --- |
| 1 | Not able to feed since birth, or stopped feeding well is dangerous |  |  |  |  |  |
| 2 | Convulsion is dangerous |  |  |  |  |  |
| 3 | Fast breathing: 60 breaths or more in one minute is dangerous |  |  |  |  |  |
| 4 | Chest indrawing is dangerous |  |  |  |  |  |
| 5 | High temperature: 37.5℃ or more is dangerous |  |  |  |  |  |
| 6 | Very low temperature: 35.4℃ or less is dangerous |  |  |  |  |  |
| 7 | Movement only when stimulated, or no movement even on stimulation is dangerous |  |  |  |  |  |
| 8 | Yellow soles is dangerous |  |  |  |  |  |
| 9 | Umbilicus red or draining pus is dangerous |  |  |  |  |  |
| 10 | Skin boils is dangerous |  |  |  |  |  |
| 11 | Eyes draining pus is dangerous |  |  |  |  |  |
| 12 | Birth within 37 weeks or birth weight <2500g is dangerous |  |  |  |  |  |
| 13 | Bluish or pale skin is dangerous |  |  |  |  |  |
| 14 | Diarrhea is dangerous |  |  |  |  |  |
| 15 | Excessive crying is dangerous |  |  |  |  |  |
| 16 | Blood in stool is dangerous |  |  |  |  |  |
| 17 | Abdominal distension is dangerous |  |  |  |  |  |
| 18 | Frequent vomiting is dangerous |  |  |  |  |  |
